# Modernized Tools for Streamlined Genetic Manipulation and Comparative Study of Wild and Diverse Proteobacterial Lineages

**DOI:** 10.1128/mBio.01877-18

**Published:** 2018-10-09

**Authors:** Travis J. Wiles, Elena S. Wall, Brandon H. Schlomann, Edouard A. Hay, Raghuveer Parthasarathy, Karen Guillemin

**Affiliations:** a Institute of Molecular Biology, University of Oregon, Eugene, Oregon, USA; b Department of Physics and Materials Science Institute, University of Oregon, Eugene, Oregon, USA; c Humans and the Microbiome Program, Canadian Institute for Advanced Research, Toronto, Ontario, Canada; University of Hawaii at Manoa; University of Connecticut; Fred Hutchison Cancer Institute

**Keywords:** Tn*7*, allelic exchange, bacterial genetics, conjugation, counterselection, domestication, genetic manipulation, microbiome, modular tools, proteobacteria, symbiosis, zebrafish

## Abstract

Correlating the presence of bacteria and the genes they carry with aspects of plant and animal biology is rapidly outpacing the functional characterization of naturally occurring symbioses. A major barrier to mechanistic studies is the lack of tools for the efficient genetic manipulation of wild and diverse bacterial isolates. To address the need for improved molecular tools, we used a collection of proteobacterial isolates native to the zebrafish intestinal microbiota as a testbed to construct a series of modernized vectors that expedite genetic knock-in and knockout procedures across lineages. The innovations that we introduce enhance the flexibility of conventional genetic techniques, making it easier to manipulate many different bacterial isolates with a single set of tools. We developed alternative strategies for domestication-free conjugation, designed plasmids with customizable features, and streamlined allelic exchange using visual markers of homologous recombination. We demonstrate the potential of these tools through a comparative study of bacterial behavior within the zebrafish intestine. Live imaging of fluorescently tagged isolates revealed a spectrum of distinct population structures that differ in their biogeography and dominant growth mode (i.e., planktonic versus aggregated). Most striking, we observed divergent genotype-phenotype relationships: several isolates that are predicted by genomic analysis and *in vitro* assays to be capable of flagellar motility do not display this trait within living hosts. Together, the tools generated in this work provide a new resource for the functional characterization of wild and diverse bacterial lineages that will help speed the research pipeline from sequencing-based correlations to mechanistic underpinnings.

## Abstract (precis)

A great challenge in microbiota research is the immense diversity of symbiotic bacteria with the capacity to impact the lives of plants and animals. Moving beyond correlative DNA sequencing-based studies to define the cellular and molecular mechanisms by which symbiotic bacteria influence the biology of their hosts is stalling because genetic manipulation of new and uncharacterized bacterial isolates remains slow and difficult with current genetic tools. Moreover, developing tools de novo is an arduous and time-consuming task and thus represents a significant barrier to progress. To address this problem, we developed a suite of engineering vectors that streamline conventional genetic techniques by improving postconjugation counterselection, modularity, and allelic exchange. Our modernized tools and step-by-step protocols will empower researchers to investigate the inner workings of both established and newly emerging models of bacterial symbiosis.

## INTRODUCTION

DNA sequencing has exposed the previously unseen diversity of symbiotic bacteria living in close contact with plants and animals throughout the biosphere (1, 2). Associations between resident bacteria and the health, development, and evolution of their hosts are being identified with incredible speed (3–7). However, the cataloging of symbiotic relationships—whether mutualistic, commensal, or pathogenic—is vastly outpacing their functional interrogation (8, 9). Characterizing the biology of symbiotic bacteria requires methods for precisely manipulating their genomes. For example, stable chromosomal insertion of genes encoding fluorescent proteins allows cellular behaviors and interactions to be directly observed within host tissues (10, 11). Additionally, gene deletion and complementation studies are essential for dissecting genetic pathways that control specific phenotypes (12). Such knock-in and knockout technologies are well established for manipulating model organisms such as Escherichia coli K-12, but many genetic approaches are incompatible with novel species or strains (13). This is largely because legacy protocols can involve outdated procedures that are difficult to use across lineages, even those that are closely related to so-called “reference” strains. Consequently, the in-depth study of most symbiotic bacteria remains out of reach.

A major bottleneck within the field of symbiosis research is that developing genetic tools for new bacterial isolates is arduous and time-consuming. This is especially burdensome for investigators aiming to manipulate multiple bacterial lineages derived from complex communities. To address this problem, we employed a collection of wild and diverse symbiotic bacteria isolated from the zebrafish intestinal microbiota (14)—which includes proteobacterial representatives of the *Vibrio*, *Aeromonas*, *Pseudomonas*, *Acinetobacter*, *Enterobacter*, *Plesiomonas*, and *Variovorax* genera—to construct a set of broadly compatible genetic tools. Proteobacteria play a major role in numerous host-microbe systems and can be drivers of both health and disease (15–24). However, genetic manipulation of proteobacterial lineages continues to be challenging due to the extreme diversity within this phylum. Therefore, molecular tools that improve the genetic tractability of this important group of bacteria will aid our understanding of their biology and how to control it.

We identified three deficiencies inherent in current genetic approaches that, if resolved, will immediately improve the genetic tractability of many bacteria. First, although conjugation is a reliable method for delivering DNA into bacteria, strategies for selecting cells carrying the transferred DNA are not easily applied across different lineages and sometimes rely on deleterious domestication steps. Second, most vectors used for making genetic manipulations cannot be customized, which restricts their versatility. And third, techniques for generating chromosomal modifications via allelic exchange often require specific selection conditions that can differ between bacterial lineages. Our solution to these shortcomings involves the rational design of genetic engineering vectors with new and updated functionalities. For DNA delivery, we developed schemes for postconjugation counterselection that avoid domestication of engineered bacteria. For customization, we designed gene expression scaffolds with interchangeable sequence elements that can be tailored to different bacterial genomes and, with these, produced several ready-made vectors for fluorescently tagging bacteria. Moreover, an extensive collection of marked zebrafish intestinal symbionts (11 strains spanning 7 genera) was generated that will aid the growing field of zebrafish microbiota research. Lastly, we devised a means of visually following homologous recombination during allelic exchange for a more tractable method of generating markerless chromosomal alterations.

We demonstrated the potential of our modernized tools to uncover new aspects of host-microbe interactions through a comparative study based on live imaging of bacterial behavior within the larval zebrafish intestine. For many strains, we observed a sharp contrast between behaviors manifested *in vivo* and those that we predicted on the basis of gene content and *in vitro* assays. Specifically, several isolates that carry genes for flagellar biogenesis and display swimming motility in soft agar became nonmotile and aggregated within the intestine. This exploratory experiment showed how tools for genetically manipulating diverse bacterial isolates facilitate broad multispecies studies capable of yielding functional insights and revealing the contextual nature of bacterial symbioses.

(This article was submitted to an online preprint archive [25]).

## RESULTS

### Temperature- and kill switch-based systems for postconjugation counterselection of donor cells.

Conjugation, or bacterial mating, is widely used for delivering DNA into bacterial cells to facilitate genetic manipulations. However, postconjugation counterselection schemes for recovering modified target cells can be inadequate in working with new and uncharacterized bacterial isolates. For example, while attempting to genetically manipulate a diverse collection of proteobacterial lineages native to the zebrafish intestine, we found that common counterselection procedures that rely on auxotrophic donor strains can be cumbersome and broadly incompatible (https://doi.org/10.6084/m9.figshare.7040267 [Text S1]). As an alternative, we derived “domesticated” target strain variants with spontaneous antibiotic resistance as a selectable marker but found that this resulted in deleterious changes in growth and behavior (https://doi.org/10.6084/m9.figshare.7040267 [Text S1]). Therefore, because of these limitations, we developed postconjugation counterselection strategies that are readily employed across different target strains independently of domestication.

We devised two plasmid-based counterselection systems that control donor cell growth by a mechanism similar to that of common suicide vectors. The first is a temperature-based system and works through a temperature-sensitive origin of replication that restricts donor cell growth in the presence of antibiotic selection at or above 37°C. Temperature-based control of plasmid replication is well established but has not been widely implemented for postconjugation counterselection despite its amenability and previous indications that it can be used in this way (26). The second system restricts donor cell growth through a genetic kill switch that, when induced, leads to the expression of toxic peptides. These two approaches differ in their modes of action and offer slightly different procedural advantages. Notably, we developed plasmid-based counterselection systems because their portability allows them to be used with different donor strains if, for example, it becomes necessary to improve conjugation efficiency or avoid the transfer of latent mobile elements (26). This feature provides an alternative to counterselection strategies that use donor strains with counterselectable traits that are chromosome based (e.g., diaminopimelic acid [DAP] auxotrophy [27]) and which, despite their effectiveness, need to be reengineered in each new donor strain. To initially test each counterselection system, we incorporated them into existing vectors commonly used for making targeted Tn*7* transposon-based chromosomal insertions in a wide range of bacterial lineages (28–33). We specifically chose Tn*7* because the *attTn7* sequence recognized by Tn*7* transposition machinery is located within the essential gene *glmS*, which encodes a glucosamine synthetase found ubiquitously across bacterial taxa (34). Consistent with previous studies (35), comparing 25 predicted *attTn7* sites from diverse lineages—including those belonging to the *Proteobacteria*, *Firmicutes*, and *Bacteroidetes*—we found that the *attTn7* sequence is widespread and conserved (https://doi.org/10.6084/m9.figshare.7040303 [Fig. S1]). Therefore, expanding the utility of preexisting Tn*7*-based tools with new counterselection systems has the potential to broadly impact the engineering of chromosomal insertions in many bacteria.

**FIG 1:**
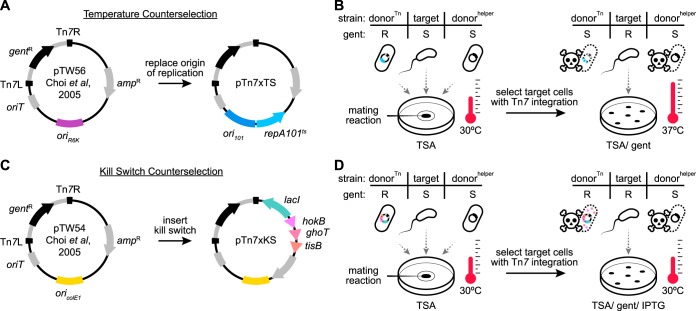
Construction and application of domestication-free counterselection systems. (A) Temperature-based counterselection was achieved by replacing the R6K origin of replication (*ori_R6K_*) of pUC18R6KT-mini-Tn7T-GM (pTW56 [28]) with the temperature-sensitive origin of replication *ori_101_*/*repA*101^ts^. Tn*7*L and Tn*7*R inverted repeats flank the Tn*7* transposon (gray stroke). *gent*^R^, gentamicin (gent) resistance gene; *amp*^R^, ampicillin resistance gene; *oriT*, origin of transfer. (B) (Left) Triparental conjugation between SM10 donor strains carrying either a temperature-sensitive Tn*7*-tagging vector (donor^Tn^) or transposase helper vector (donor^helper^) and a *Vibrio* target strain. The gentamicin phenotype of each strain is indicated as resistant (R) or sensitive (S). Mating reactions were incubated at 30°C on a filter disc on a trypic soy agar (TSA) plate. (Right) Postconjugation counterselection of donor cells was done on TSA/gent plates at 37°C. (C) Kill switch-based counterselection was achieved by inserting a LacI-regulated toxin array, comprised of the genes *hokB*, *ghoT*, and *tisB*, into the backbone of pUC18T-mini-Tn7T-GM (pTW54 [28]). *ori_ColE1_*, high-copy-number origin of replication. (D) (Left) Triparental conjugation performed as described for panel B, except that donor^Tn^ carried a kill switch Tn*7*-tagging vector. (Right) Postconjugation counterselection of donor cells was done on TSA/gent/IPTG plates at 30°C.

Temperature-based counterselection was achieved by replacing the R6K origin of replication of Tn*7*-tagging vector pUC18R6KT-mini-Tn7T-GM (pTW56) with the temperature-sensitive origin of replication *ori_101_*/*repA*101^ts^ (36) (Fig. 1A). The resulting vector, pTn7xTS (temperature sensitive), mediates temperature-dependent growth of E. coli SM10 (https://doi.org/10.6084/m9.figshare.7040210 [Fig. S2]). At the permissive temperature of 30°C, SM10/pTn7xTS grew on rich media in the presence of antibiotic selection. At the restrictive temperature of 37°C, the vector was unable to be maintained, leading to loss of antibiotic resistance and a drop in viability by several orders of magnitude. In the context of an example Tn*7*-tagging protocol, conjugation between two SM10 donor strains and a *Vibrio* target strain was performed at 30°C without antibiotic selection (Fig. 1B, left). The SM10 donors carried either pTn7xTS (donor^Tn^) or the transposase-encoding helper plasmid pTNS2 (donor^helper^). At this point in the procedure, only the donor^Tn^ strain was resistant to the selective antibiotic being used, which in this scenario was gentamicin. Modified *Vibrio* cells harboring a chromosomally integrated Tn*7* transposon and the gentamicin resistance gene that it encodes were then selected by plating the mating mixture in the presence of gentamicin at 37°C (Fig. 1B, right). The donor^Tn^ strain was counterselected because it was unable to maintain plasmid-based resistance at 37°C, whereas the donor^helper^ strain remained sensitive to gentamicin.

A strength of temperature-based counterselection is that it is technically simple, requiring only a shift in growth temperature, but it is limited to target strains that can grow at 37°C. This is problematic for several bacterial lineages native to zebrafish as well as other ectotherms, such as stickleback or fruit flies, which cannot survive at temperatures above the growth temperature of their host (in these cases, ≤30°C). Therefore, we developed a second strategy based on an inducible kill switch that functions independently of the growth temperature.

The kill switch that we designed consists of two elements: a constitutively expressed *lacI* gene encoding the *lac* repressor and a synthetic operon containing three E. coli-derived genes encoding toxic peptides—HokB, GhoT, and TisB—placed under the control of the P_tac_ LacI-repressible promoter (https://doi.org/10.6084/m9.figshare.7040207 [Fig. S3A]) (37–42). Upon induction by the allolactose analogue IPTG (isopropyl-β-d-thiogalactopyranoside), these toxic peptides act to disrupt the proton-motive force within donor cells, leading to impaired ATP synthesis and death. We built this kill switch counterselection system into the backbone of the Tn*7*-tagging vector pUC18T-mini-Tn7T-GM (pTW54), producing pTn7xKS (kill switch) (Fig. 1C). In the presence of antibiotic selection and IPTG, pTn7xKS was capable of inhibiting SM10 growth by up to 4 orders of magnitude (https://doi.org/10.6084/m9.figshare.7040207 [Fig. S3B]). Of note, an initial kill switch prototype carrying a single toxin gene was less effective, indicating either that the use of multiple toxins increases potency or that toxin redundancy limits mutational escape. In the context of a Tn*7*-tagging scenario, kill switch-based counterselection was carried out in much the same way as temperature-based counterselection, except that selection of modified target cells was done on media containing IPTG at a growth temperature suitable for the target strain being used (Fig. 1D).

### Construction of rationally designed gene expression scaffolds and their chromosomal insertion into diverse proteobacterial lineages using domestication-free counterselection.

To test the effectiveness of our domestication-free counterselection systems, we employed them to integrate genetically encoded fluorescent proteins into the chromosome of various uncharacterized zebrafish bacterial symbionts. However, we found that many available gene expression constructs are inflexible and inadequately designed. Vectors often contained extraneous DNA sequences and had few to no options for customizing important sequence motifs. The ability to customize expression constructs is critical when working with lineages that differ in, for instance, optimal promoter sequences or ribosome binding sites. Therefore, we first addressed the need for standardized expression constructs by rationally designing a modular gene expression scaffold.

**FIG 2:**
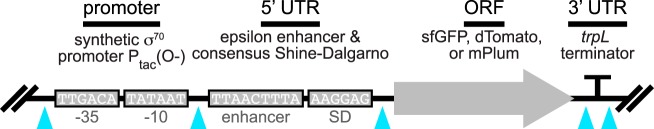
Gene expression scaffold design features. Each interchangeable element is flanked by restriction sites (cyan arrowheads). Promoter, constitutively active P_tac_ promoter without lac operator sequence (O-) driving transcription; the 5′ untranslated region (UTR), epsilon enhancer sequence, and consensus ribosome binding site (i.e., Shine-Dalgarno sequence) promote strong translation. ORF, open reading frame (encoding a single fluorescent protein); 3′ UTR, *trpL* attenuator sequence terminating transcription.

An expression scaffold containing four interchangeable elements—a promoter, 5′ and 3′ untranslated regions (UTR), and an open reading frame (ORF)—was built into the multiple-cloning site (mcs) of pGEN-mcs (43), producing pXS (expression scaffold) (Fig. 2). pGEN-mcs was chosen to house the expression scaffold because it enables rapid prototyping of scaffold parts in E. coli, which, like many zebrafish bacterial symbionts, is a member of the *Gammaproteobacteria* and shares basic genetic control elements. Restriction sites underlie the modular architecture of the scaffold, allowing sequence motifs to be replaced individually or all together (https://doi.org/10.6084/m9.figshare.7040246 [Fig. S4A]). As initially built, a minimal P_tac_ promoter without the *lac* operator sequence, which avoids potential interference from an endogenously encoded *lac* repressor, was used to achieve constitutive transcription. A synthetic 5′ UTR containing both an epsilon enhancer sequence and ribosome binding site controls translation (44, 45). The 3′ UTR, which was originally present within pGEN-mcs, contains a *trpL* attenuator sequence for transcriptional termination (46). Lastly, three different ORFs encoding the fluorescent proteins superfolder green fluorescent protein (sfGFP) (47), dTomato (48), and mPlum (49) were each used to produce three separate expression scaffold variants, pXS-sfGFP, pXS-dTomato, and pXS-mPlum. After assembly, each scaffold was subcloned into Tn*7*-tagging vectors with either temperature (pTn7xTS) or kill switch (pTn7xKS) counterselection systems (https://doi.org/10.6084/m9.figshare.7040246 [Fig. S4B and C]).

Tn*7*-tagging vectors equipped with rationally designed expression scaffolds were next used to carry out chromosomal tagging of the zebrafish intestinal symbiont Vibrio cholerae ZWU0020 as outlined in Fig. 1B and D. Unlike previous attempts (https://doi.org/10.6084/m9.figshare.7040267 [Text S1]), domestication-free tagging of *Vibrio* ZWU0020—using either temperature or kill switch counterselection systems—preserved this strain’s normal physiology (https://doi.org/10.6084/m9.figshare.7040216 [Fig. S5]). To demonstrate the compatibility of these tools across zebrafish symbiont lineages, we tagged a collection of 11 strains that includes representatives from 2 proteobacterial classes, 5 orders, and 7 genera (https://doi.org/10.6084/m9.figshare.7040249 [Table S1]). Multiple variants that express *sfGFP*, *dTomato*, or *mPlum* were generated for many of the isolates. We also verified that domestication-free counterselection systems can be used to manipulate species not associated with the zebrafish intestinal microbiota, including a human-derived strain of E. coli (HS) (50), the leech symbiont Aeromonas veronii HM21 (51), and an environmental isolate of Shewanella oneidensis (MR-1) (52) (https://doi.org/10.6084/m9.figshare.7040249 [Table S1]).

### Streamlining allelic exchange by visualizing homologous recombination events using a fluorescent tracker.

Allelic exchange is a versatile homologous recombination technique for making targeted genetic knock-ins and knockouts in bacteria (53–55). To extend the utility of our domestication-free counterselection systems, we incorporated them into currently available vectors that are used for mediating allelic exchange. These updates facilitated the domestication-free engineering of gene deletions in several uncharacterized symbiotic bacteria. However, not all bacteria tested could be successfully manipulated using current allelic exchange protocols, highlighting another breakdown in the compatibility.

Allelic exchange involves two successive homologous events of recombination between an allelic exchange vector and the bacterial chromosome. The crux of allelic exchange is isolating rare unmarked mutant cells from the large populations of heterozygous intermediates (known as merodiploids) that arise after the vector integrates into the chromosome during the first recombination step. A longstanding strategy for recovering variants that have undergone the second recombination, which results in vector loss, works by restricting merodiploid growth. This is typically done by expressing a gene called *sacB* located within the allelic exchange vector backbone that confers growth inhibition in the presence of sucrose (56). Although widely used, *sacB* counterselection of merodiploids does not always work and can be difficult to troubleshoot when it fails. We experienced these shortcomings while attempting to delete a gene associated with the chemotactic behavior of a zebrafish symbiont, Vibrio fluvialis ZOR0035, employing the commonly used *sacB*-based allelic exchange vector pDMS197 (57). *Vibrio* ZOR0035 merodiploids are refractory to *sacB* counterselection, which made it impossible to isolate cells with the desired mutation. We surmise that counterselection fails in some bacterial lineages because the expression or activity of the levansucrase enzyme encoded by *sacB*, which synthesizes high-molecular-weight fructose polymers, is inadequate. To overcome lineage-specific limitations of *sacB* counterselection, we developed a more tractable strategy based on visual markers.

**FIG 3:**
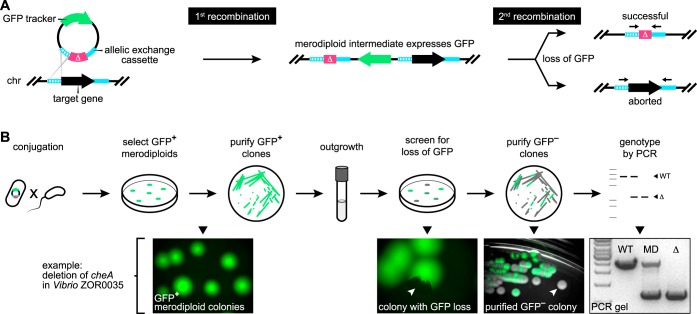
Performing allelic exchange with a fluorescent merodiploid tracker. (A) Outline of recombination events during allelic exchange with a fluorescent tracker. An allelic exchange vector is depicted that expresses GFP and carries a cassette comprised of a mutant allele (Δ, magenta) flanked by regions (hashed and solid cyan strokes) homologous to regions flanking a target gene located on the bacterial chromosome (chr). The first recombination event—which randomly occurs between either homology region—integrates the vector into the chromosome, producing a GFP-expressing merodiploid. The second recombination event results in GFP loss. If it occurs in the unused homology region (i.e., the “solid” region in this scenario), then allelic exchange is successful. If it occurs in the same region (i.e., the “hashed” region), the original wild-type locus is restored. Black arrows shown above the final allelic exchange products denote primer annealing sites for PCR-based genotyping depicted in panel B. (B) The top row illustrates the procedural steps of allelic exchange performed using a fluorescent merodiploid tracker. The bottom row shows example images acquired during the engineering of a gene deletion in *Vibrio* ZOR0035. White arrowheads indicate colonies with partial or complete loss of GFP expression. WT, wild-type *Vibrio* ZOR0035; MD, merodiploid; Δ, Δ*cheA* mutant.

Our solution uses GFP to track the merodiploid status of target cells (Fig. 3A). In this way, the initial recombination step generates GFP-positive merodiploid populations that can be readily screened for cells where the second recombination step has occurred, producing GFP-negative mutants (i.e., instances of “successful” allelic exchange), which typically arise at frequencies equal to those seen with wild-type revertants (i.e., instances of “aborted” allelic exchange) (Fig. 3A). To test the feasibility of this approach, we revisited the engineering of a gene deletion in *Vibrio* ZOR0035. A constitutively expressed GFP gene was inserted into the backbone of a prototype pDMS197 vector containing a kill switch counterselection system and an allelic exchange cassette targeting the chemotaxis gene *cheA*. As outlined in Fig. 3B, the GFP marked allelic exchange vector was delivered into *Vibrio* ZOR0035 via conjugation. GFP-positive merodiploids harboring an integrated copy of the allelic exchange vector were isolated and purified. Of note, we empirically determined that the kill switch toxins did not interfere with merodiploid growth in several different bacterial species, indicating either that they have restricted activity and are lethal only to E. coli donor cells or that they fail to reach toxic levels when expressed from a single chromosomal locus. Next, populations of merodiploids were expanded in liquid culture and plated on nonselective media at a density that allowed discrete colonies to form. Colonies exhibiting GFP loss were purified to obtain isogenic clones, and putative mutants were genotyped by PCR. Genotyping was done using PCR primers flanking *cheA*, yielding a single large amplification product when *cheA* was present and a smaller-sized product for the mutant allele. Heterozygous merodiploids produce both products.

Our visual merodiploid tracking strategy proved extremely efficient and straightforward to perform. We were able to screen thousands of merodiploid colonies for second recombination events in a matter of minutes using a fluorescence stereomicroscope. Interestingly, the manner in which GFP-negative cells arose revealed an additional advantage of our approach over conventional *sacB*-based counterselection. From five replicate merodiploid cultures, we found that second recombination events at the *cheA* locus in *Vibrio* ZOR0035 were indeed rare, occurring with a median frequency of 1.05 × 10^−3^ (https://doi.org/10.6084/m9.figshare.7040222 [Fig. S6]) (Table 1). However, only one of the five cultures produced colonies with complete loss of GFP. These GFP-negative colonies represent recombination events that occurred during growth in liquid media prior to plating. By contrast, we found that the majority of second recombination events across all cultures arose from merodiploid cells as GFP-negative sectors within GFP-positive merodiploid colonies after plating (Fig. 3B and Table 1). This distinction is important because for *sacB*-based counterselection to work, *sacB* must be removed via the second recombination event prior to plating; otherwise, the growth of merodiploid cells is inhibited. Therefore, not only did our approach allow us to successfully engineer a targeted gene deletion in a bacterial strain that was genetically intractable using current methods, it also enhanced the likelihood of recovering the rare cells that had undergone a second recombination event.

**TABLE 1:** Frequency of second recombination events in *V. fluvialis* ZOR0035 *cheA* merodiploids

Replicate culture^*a*^	No. of colonies screened^*b*^	Total no. (frequency) of colonies with complete GFP loss	Total no. (frequency) of colonies with partial GFP loss
1	952	0 (0)	1 (1.05 × 10^−3^)
2	11,627	0 (0)	4 (3.44 × 10^−4^)
3	962	2 (2.08 × 10^−3^)	2 (2.08 × 10^−3^)
4	853	0 (0)	1 (1.17 × 10^−3^)
5	8,833	0 (0)	3 (3.40 × 10^−4^)

a Each replicate culture represents an independently grown culture derived from the same *V. fluvialis* ZOR0035 *cheA* merodiploid clone.

b Merodiploid cells were spread onto multiple TSA plates at a density that allowed discrete colonies to form and were screened using a fluorescence stereomicroscope.

### Markerless gene deletion and complementation with modernized engineering vectors.

**FIG 4:**
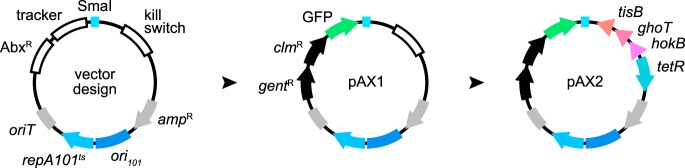
Rational design of customizable allelic exchange vectors. “vector design” illustrates the vector architecture. Features include customizable molecular scaffolds for holding antibiotic selection markers (Abx^R^), a merodiploid tracker, a single Smal restriction site for insertion of allelic exchange cassettes, and an option for kill switch-based counterselection of donor cells. pAX1 was initially constructed and carries two antibiotic selection markers encoding resistance to gentamicin (*gent*^R^) and chloramphenicol (*clm*^R^) along with a GFP tracker. pAX2 was derived via the insertion of a tet-inducible kill switch. *oriT*, origin of transfer; *ori_101_*/*repA*101^ts^, temperature-sensitive origin of replication; *amp*^R^, ampicillin resistance gene.

To make the genetic toolkit for manipulating wild and diverse bacterial isolates more complete, we combined the approaches described thus far to construct a set of adaptable allelic exchange vectors that further improved the tractability of markerless genetic alterations. These modernized vectors incorporate fluorescent merodiploid tracking and domestication-free counterselection systems within a highly customizable plasmid architecture (Fig. 4, “vector design”). Molecular scaffolds designed for holding antibiotic selection markers, fluorescent trackers, and a counterselection kill switch were built into a pUC-derived vector backbone that had a temperature-sensitive origin of replication and a single blunt restriction site for straightforward insertion of allelic exchange cassettes. This modular design allows virtually every functional element to be customized (https://doi.org/10.6084/m9.figshare.7040219 [Fig. S7]).

Two allelic exchange vectors were generated, pAX1 and pAX2 (allelic exchange), which differ in their domestication-free counterselection systems (Fig. 4). Both vectors mediate temperature-based counterselection of SM10 donor cells, but pAX2 also contains a TetR-regulated kill switch that can be induced by anhydrotetracycline (https://doi.org/10.6084/m9.figshare.7040225 [Fig. S8]). Notably, the dual-temperature/kill switch counterselection activity of pAX2 is potent, reducing SM10 viability by over 5 orders of magnitude. Two resistance markers for gentamicin and chloramphenicol were included to give pAX1 and pAX2 more-immediate compatibility with different target strains.

**FIG 5:**
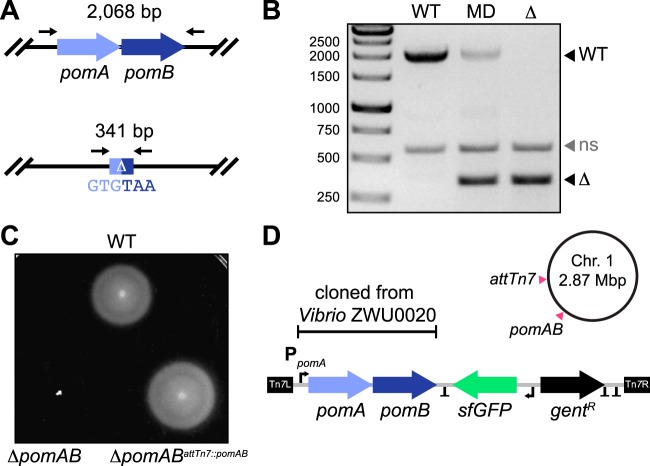
Gene deletion and complementation with modernized engineering vectors. (A) (Top) Wild-type *pomAB* locus in *Vibrio* ZWU0020. (Bottom) Result of markerless *pomAB* deletion via allelic exchange. Black arrows mark approximate primer annealing sites for genotyping, and the size of each amplification product is indicated. (B) Agarose gel showing PCR-based genotyping of the wild-type strain (WT), a merodiploid strain (MD), and a Δ*pomAB* (Δ) mutant. Migration distances of WT and mutant alleles are indicated. ns, nonspecific amplification product. (C) Swim motility of the WT strain, the Δ*pomAB* mutant, and the complemented Δ*pomAB^attTn7^*^::^*^pomAB^* variant in 0.2% tryptic soy agar at 30°C. (D) Shown is a schematic of the Tn*7* transposon from pTn7xTS-sfGFP used for complementation, which was modified to carry the native *pomAB* locus of *Vibrio* ZWU0020. Also depicted are the relative positions where the *pomAB* genes were deleted and reintroduced at the Tn*7* insertion site (*attTn7*) on chromosome 1 of *Vibrio* ZWU0020. T, transcriptional terminators; Tn*7*L and Tn*7*R, Tn*7* inverted repeats; P_pomA_, native *pomA* promoter; *gent*^R^, gentamicin resistance gene; sfGFP, fluorescent tag.

We next used our newly constructed pAX vectors to engineer markerless gene deletions. For this proof of concept, we designed an allelic exchange cassette to delete two neighboring genes in *Vibrio* ZWU0020, *pomA* and *pomB*, which encode the polar flagellar motor. After inserting the cassette into an early but functionally equivalent version of pAX1 (see Materials and Methods), GFP-positive merodiploids were generated and isolated as previously described and screened for loss of GFP expression (https://doi.org/10.6084/m9.figshare.7040228 [Fig. S9], https://doi.org/10.6084/m9.figshare.7040231 [Fig. S10], and https://doi.org/10.6084/m9.figshare.7040312 [Data Set S1]). Mutants harboring the desired mutation, which fused the start codon of *pomA* with the stop codon of *pomB*, were confirmed by PCR (Fig. 5A and B). As anticipated, ZWU0020 Δ*pomAB* exhibited complete loss of swimming motility in soft agar (Fig. 5C, bottom left) without overt growth defects in liquid media (https://doi.org/10.6084/m9.figshare.7040228 [Fig. S9]). To demonstrate the cross-lineage compatibility of these tools, we successfully employed pAX2 to create a similar markerless deletion of a homologous *pomAB* locus in another zebrafish symbiont, Aeromonas veronii ZOR0001 (https://doi.org/10.6084/m9.figshare.7040240 [Fig. S11], https://doi.org/10.6084/m9.figshare.7040237 [Fig. S12], and https://doi.org/10.6084/m9.figshare.7040312 [Data Set S1]). Notably, in screening *Vibrio* ZWU0020 and *Aeromonas* ZOR0001 merodiploid colonies, we observed many different patterns of GFP loss (https://doi.org/10.6084/m9.figshare.7040234 [Fig. S13]). Remarkably, even when mutant cells resided within miniscule GFP-negative patches, isolating them often required only a single colony purification step. The ability to identify and recover mutant cells with such sensitivity highlights the robustness of our visual screening approach.

To confirm that mutant phenotypes are the result of targeted genetic disruptions and not polar effects or unintended consequences of chromosomal manipulation, complementation must also be performed (12). Therefore, we wanted to demonstrate how our domestication-free Tn*7*-tagging vectors could be employed to complement the ZWU0020 Δ*pomAB* mutant. The *pomAB* locus of ZWU0020, including the native *pomA* promoter, was PCR amplified and inserted within the Tn*7* transposon of tagging vector pTn7xTS-sfGFP (Fig. 5D). Chromosomal insertion of this construct at the *attTn7* site of ZWU0020 Δ*pomAB* fully restored wild-type motility, thus confirming that it was solely disruption of these genes that caused the loss of motility in the mutant (Fig. 5C, bottom right).

### Probing the *in vivo* behavior of diverse bacterial symbionts reveals divergent genotype-phenotype relationships.

**FIG 6:**
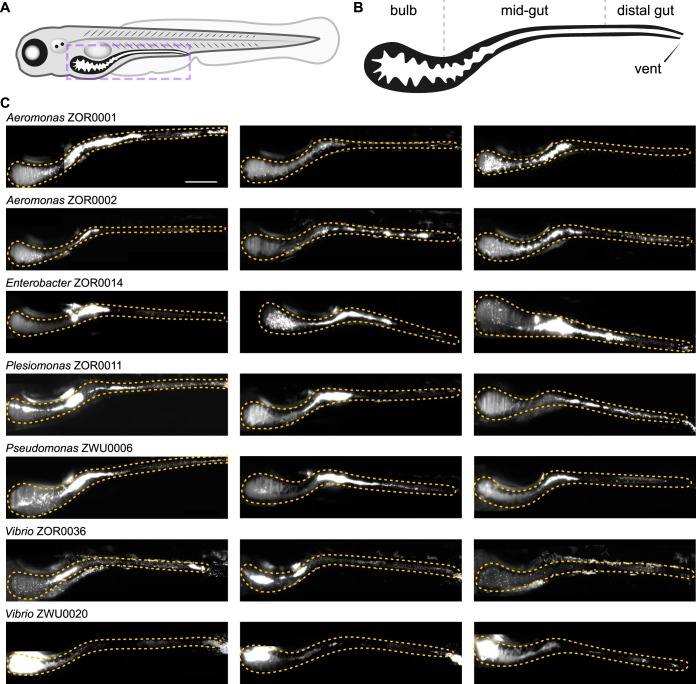
Intestinal colonization patterns and growth modes of zebrafish symbionts. (A) Cartoon diagram of a 5-day-old larval zebrafish. The purple dashed box outlines the region imaged in panel C. (B) Diagram showing the boundaries of the bulb, midgut, and distal gut within the larval intestine. The estimated bulb-to-midgut boundary is located where the bulb begins to become patently narrow. The midgut-to-distal-gut boundary is approximately located where intestinal epithelial cells begin transitioning to a more colonic epithelial cell type (59). (C) Three maximum intensity projections of three-dimensional (3D) image stacks acquired by light sheet fluorescence microscopy for the indicated bacterial strains. An orange dotted outline marks the intestine in each image. Scale bar: 200 μm.

A major challenge in microbiota research is understanding how the functional potential of symbiotic bacteria, commonly inferred through DNA sequencing methods, relates to the actual expression of phenotypes during host association. Determining the fidelity of this connection is important because it affects the interpretation of phylogenetic and metagenomic analyses frequently used to describe host-microbe interactions. To illustrate how the tools developed in this work facilitate the investigation of genotype-phenotype relationships, we compared the behaviors of seven fluorescently tagged symbionts within the zebrafish intestine using light sheet fluorescence microscopy (10, 58). The strains chosen included *Aeromonas* ZOR0001, *Aeromonas* ZOR0002, *Enterobacter* ZOR0014, *Plesiomonas* ZOR0011, *Pseudomonas* ZWU0006, *Vibrio* ZOR0036, and *Vibrio* ZWU0020. Prior to imaging, bacteria were associated individually with 4-day-old, germfree larval zebrafish for 24 h. Monoassociation provides unrestricted access to the intestinal environment free of competition with resident microbiota, allowing interactions between a single symbiont and its host to be studied in isolation. For each strain, real-time two-dimensional movies and three-dimensional images spanning the entire volume of the larval intestine were acquired from three separate hosts (Fig. 6) (https://doi.org/10.6084/m9.figshare.c.4141163 [Movies S1 to S7]). From these data, different population structures are readily identified that can be distinguished by their biogeography and dominant growth mode (i.e., planktonic versus aggregated). Additional features of each strain’s population, including estimated abundance, are provided at https://doi.org/10.6084/m9.figshare.7040255 (Table S2).

The locations of bacterial populations could be broadly classified as being distributed across one of two regions, namely, either the proximal gut (referred to here as the “bulb”) or the midgut, which we approximate based on anatomical landmarks indicated in Fig. 6B (59, 60). For most strains (e.g., *Aeromonas* ZOR0001, *Aeromonas* ZOR0002, *Enterobacter* ZOR0014, *Plesiomonas* ZOR0011, *Pseudomonas* ZWU0006, and *Vibrio* ZOR0036), the bulk of the population localizes throughout the midgut (Fig. 6C). By contrast, populations of *Vibrio* ZWU0020 reside within the proximal portion of the bulb, consistent with previous findings (Fig. 6C) (10). Planktonic and aggregated growth modes were observed within each population, but the proportion of cells in each mode tended to be strain specific. For example, at one extreme, populations of *Vibrio* ZWU0020 are almost entirely comprised of highly motile planktonic cells, which is more discernible in movies because of this strain’s high population density and fast movement (https://doi.org/10.6084/m9.figshare.7040309 [Movie S1]). At the opposite extreme, *Enterobacter* ZOR0014 forms large multicellular aggregates (Fig. 6C) (https://doi.org/10.6084/m9.figshare.7040276 [Movie S2]). The remaining strains produce populations with intermediate mixtures of planktonic and aggregated cells (Fig. 6C).

**FIG 7:**
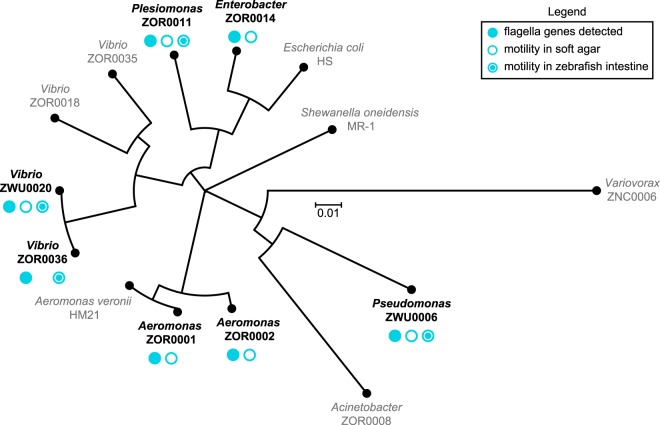
Phylogenetic relatedness and summary of motility phenotypes. Shown is a phylogenetic tree generated using nucleotide sequences of the 16S rRNA gene from all strains manipulated in this study. Strains used for live imaging are indicated in bold black type. Symbols denote whether genes associated with flagellar motility were detected by genomic analysis and if motility behaviors were observed *in vitro* and/or *in vivo*.

Surprisingly, comparing symbiont behaviors within living hosts revealed discrepancies between the motility phenotype of strains *in vivo* and their predicted capacity for motility based on phylogenetic relatedness, genome sequence, and *in vitro* assays (Fig. 7) (https://doi.org/10.6084/m9.figshare.7040243 [Fig. S14] and https://doi.org/10.6084/m9.figshare.c.4141163 [Movies S1 to S7]). Each strain examined carries genes for flagellum biogenesis and displays free-swimming motility in liquid media. All strains were also found to swim in soft agar, with the exception of *Vibrio* ZOR0036. Yet despite these attributes, motile individuals were not observed within intestinal populations of *Enterobacter* ZOR0014, *Aeromonas* ZOR0001, and *Aeromonas* ZOR0002 (https://doi.org/10.6084/m9.figshare.c.4141163 [Movies S1 to S4]). In a previous study involving *Aeromonas* ZOR0001 (10), motile cells were detected, but their occurrence was extremely rare. In addition, although *Vibrio* ZOR0036 exhibited little motility in soft agar, it gave rise to a considerable number of highly motile cells *in vivo* (https://doi.org/10.6084/m9.figshare.7040282 [Movie S5]); however, its overall motility phenotype is muted compared to that of the closely related *Vibrio* ZWU0020 strain (Fig. 7) (https://doi.org/10.6084/m9.figshare.7040243 [Fig. S14] and https://doi.org/10.6084/m9.figshare.7040309 [Movie S1]). The results from our comparative study begin to reveal the highly contextual nature of bacterial symbioses, which would be difficult to capture using *in silico* and *in vitro* approaches alone or solely by examining individual reference isolates.

## DISCUSSION

### Utility of the tools produced by this work.

**FIG 8:**
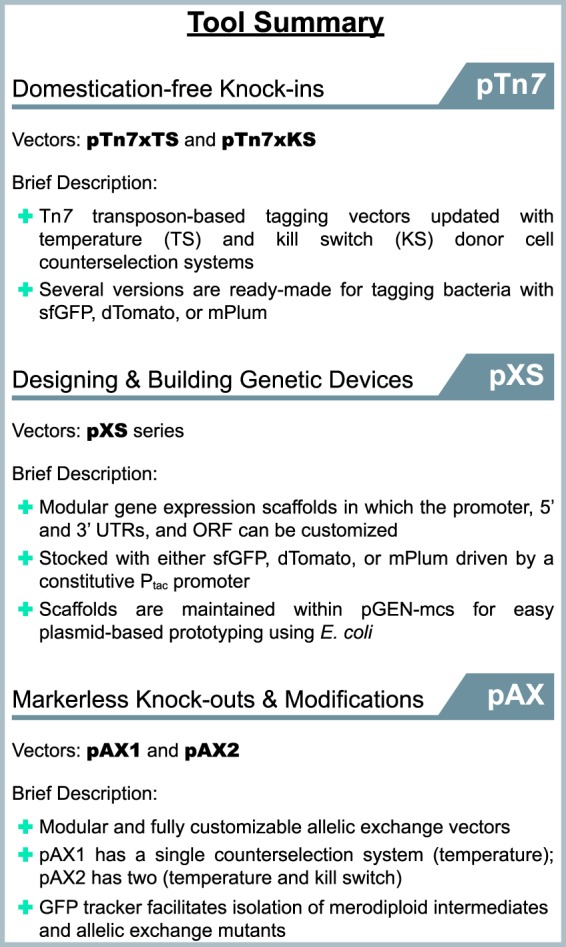
Summary of the genetic tools described in this work. The functions and features of each engineering vector are briefly summarized. The guide is organized based on technique or intended use.

Since the discovery of bacterial transformation almost a century ago (61), microbiologists have generated an extensive collection of molecular tools for genetically manipulating bacteria; however, many approaches have been optimized for only a small number of species and reference strains. The narrow compatibility of genetic tools is a significant limitation for researchers looking to manipulate diverse bacterial lineages isolated from complex microbiota. Moreover, for those working with novel and uncharacterized bacterial isolates or new to performing genetic manipulations altogether, developing basic molecular tools and protocols represents a major obstacle. We have addressed these issues by constructing standardized engineering vectors that streamline the process of making genetic knock-ins and knockouts across a broad range of bacterial lineages. These tools are summarized in Fig. 8, and their features and limitations are discussed below.

### Minimizing domestication of wild bacterial isolates.

Laboratory domestication is commonly used to improve genetic tractability or help discern specific bacterial strains within complex polymicrobial environments (e.g., the vertebrate intestine, water, or soil). However, domestication can alter bacterial physiology and impair behaviors involved in host colonization (https://doi.org/10.6084/m9.figshare.7040267 [Text S1]) (62–64). Exemplifying this, some laboratory-derived lineages of Vibrio fischeri, which is the light organ symbiont of the Hawaiian bobtail squid (Euprymna scolopes), have lost the ability to utilize glycerol as a carbon source (possibly the unintended result of prolonged growth on rich media) and display attenuated host colonization (as a consequence of antibiotic-based domestication) (64). The accurate modeling of symbiotic interactions requires careful attention to preserving natural symbiont biology. The temperature- and kill switch-based counterselection systems of the Tn*7* tagging vectors pTn7xTS and pTn7xKS (Fig. 1) and of the novel allelic exchange vectors pAX1 and pAX2 (Fig. 4) offer domestication-free strategies for manipulating various symbiotic bacterial lineages. Although our strategies for domestication-free counterselection were used in the context of conjugation, the engineering vectors that we constructed can just as easily be introduced via alternative DNA delivery strategies such as electroporation or natural competence. Moreover, both temperature- and kill switch-based counterselection systems can be used with target strains that have higher optimal growth temperatures than those in this study (e.g., ≥37°C) since genetic modifications based on Tn*7* and allelic exchange are chromosomal and target strains are thus not required to carry engineering vectors episomally.

### Broad utility through modularity.

The incredible genetic and phenotypic diversity of bacterial species challenges the cross-lineage compatibility of genetic tools. We rationally designed tools with modular architectures to make it easier to adapt and expand on original designs. For example, starting with the expression scaffolds within the pXS series of vectors, we have engineered more-elaborate genetic devices, including reporters of gene expression and genetic circuits. Additionally, the modularity of pXS expression scaffolds is advantageous when genetic parts need to be generated *de novo*. For example, libraries in which constructs contain a single variable motif (e.g., a promoter or ribosome binding site) can be readily assembled and screened for optimal activity. While the flexibility of our expression scaffolds is conducive to innovation, as built, they serve as a starter kit of essential genetic parts with immediate utility for stably tagging bacteria. We also incorporated modularity into the design of the pAX1 and pAX2 allelic exchange vectors (Fig. 4) (https://doi.org/10.6084/m9.figshare.7040219 [Fig. S7]). Several elements within these vectors can be customized, including the antibiotic resistance genes, fluorescent merodiploid tracker, and kill switch. Notably, pAX vectors can be used as an alternative to Tn*7*-based strategies for creating genetic knock-ins. Tn*7* is restricted to bacteria with an *attTn7* insertion site, and, in some cases, Tn*7* tagging can interfere with neighboring genes, which we found occurs in *Variovorax* ZNC0006 (https://doi.org/10.6084/m9.figshare.7040249; see note in Table S1). The pAX vectors provide an adaptable tagging solution that allows genetic insertions to be made throughout the chromosome. In total, tailorable tools offer a way of tuning and customizing functionality to increase the experimental potential of bacterial isolates. Although the tools and strategies that we developed are primarily built for genetically manipulating diverse gammaproteobacterial lineages, their modularity will make it possible to adapt them to other proteobacterial lineages as well as to bacteria from other phyla.

### Streamlining genetic manipulations with visual screening.

To improve the tractability of allelic exchange, we used GFP to visually track recombination events (Fig. 3). This simple update proved extremely powerful. It allowed merodiploids to be confidently identified and isolated while final mutant derivatives could be screened for with incredible sensitivity, including sometimes being found as small subpopulations within merodiploid colonies growing on an agar plate (Fig. 3) (https://doi.org/10.6084/m9.figshare.7040228 [Fig. S9], https://doi.org/10.6084/m9.figshare.7040240 [Fig. S11], and https://doi.org/10.6084/m9.figshare.7040234 [Fig. S13]). The successful manipulation of a previously intractable bacterium (i.e., *Vibrio* ZOR0035) highlights the utility of this approach. Although conventional selection schemes (e.g., based on *sacB*) are adept at recovering mutants that arise at low frequencies due to rarely occurring recombination events, their use is contingent on specific conditions that can be difficult to translate between species or strains. By contrast, our visual screening approach operates freely across lineages and differs from selections in that it allows the progression of recombination events to be more precisely monitored. As a result, the engineering and isolation of bacterial mutants are more attainable. We expect that the visual merodiploid tracking feature of pAX1 and pAX2 will be compatible with bacterial taxa beyond those used in this study. The primary constraints of the current pAX vector designs are the use of fluorescent proteins as trackers and the specific promoter sequences driving their expression. However, because the pAX vectors are fully customizable, appropriate promoters and alternative trackers not based on fluorescence, for example, beta-galactosidase, luminescent proteins, or pigments, can be easily incorporated.

### New avenues for the investigation of bacterial symbioses.

The tools and approaches developed in this work will advance the investigation of bacterial symbioses in two important ways. First, their technical flexibility facilitates the exploration of previously uncharacterized lineages, which is critical for constructing a more comprehensive picture of the functional diversity of symbiotic bacteria. And second, they make conducting multilineage comparative studies more feasible, which allows previously unrecognized interactions to be identified. These experimental avenues have the power to clarify many properties of complex symbiotic associations, such as those involving the human intestinal microbiota (65, 66), which can be extremely variable and remain unexplained.

Illustrating the potential of studying new bacterial isolates and performing comparative studies, we exposed several uncharacterized interactions by probing the spatial colonization patterns and behaviors of multiple lineages native to the zebrafish intestine. Among the most apparent results, we found discordance between predicted and observed motility phenotypes *in vivo*. All strains examined carried flagellar genes and tended to be highly motile *in vitro*, and yet several of them (e.g., *Enterobacter* ZOR0014, *Aeromonas* ZOR0001, and *Aeromonas* ZOR0002) displayed no obvious motility during intestinal colonization (https://doi.org/10.6084/m9.figshare.c.4141163 [Movies S2 to S4]). By contrast, some strains, most notably, *Vibrio* ZWU0020, robustly sustained motility *in vivo* (https://doi.org/10.6084/m9.figshare.7040309 [Movie S1]). There are several possible explanations for these behaviors that are not necessarily mutually exclusive. They may be the result of bacteria responding to the intestinal environment and executing strain- or lineage-specific colonization strategies. On the other hand, bacteria could be differentially susceptible to some form of host-mediated motility interference, which has recently been documented in mouse models (67, 68). Together, our observations highlight the limitations of *in silico*- and *in vitro*-based studies and underscore the importance of tools that are utilizable for characterizing diverse bacterial lineages in the context of their native host-associated environments.

## MATERIALS AND METHODS

### Supplemental material.

### Animal care.

All experiments with zebrafish were done in accordance with protocols approved by the University of Oregon Institutional Animal Care and Use Committee and following standard protocols (69).

### Gnotobiology.

Wild-type (AB × TU strain) zebrafish were derived germfree (GF) and colonized with bacterial strains as previously described (70) with slight modifications. Briefly, fertilized eggs from adult mating pairs were harvested and incubated in sterile embryo media (EM) containing ampicillin (100 μg/ml), gentamicin (10 μg/ml), amphotericin B (250 ng/ml), tetracycline (1 μg/ml), and chloramphenicol (1 μg/ml) for ∼6 h. Embryos were then washed in EM containing 0.1% polyvinylpyrrolidone-iodine followed by EM containing 0.003% sodium hypochlorite. Sterilized embryos were distributed into T25 tissue culture flasks containing 15 ml sterile EM at a density of one embryo per milliliter and incubated at 28 to 30°C prior to bacterial colonization. Embryos were sustained on yolk-derived nutrients and were not fed during experiments. For bacterial monoassociation studies, bacterial strains were grown overnight in LB liquid media with shaking at 30°C and were prepared for inoculation by pelleting 1 ml of culture for 2 min at 7,000 × *g* and washing once in sterile EM. Bacterial strains were individually added to the water column of single flasks containing 4-day-old larval zebrafish at a final density of ∼10^6^ bacteria/ml. Bacterial colonization patterns were assessed 24 h later by live imaging of three separate 5-day-old zebrafish hosts per bacterial strain. Three animals were determined to be adequate for capturing the general colonization features of each bacterial strain on the basis of at least two previous independent qualitative assessments of colonization patterns.

### Bacterial strains and culture.

All wild and recombinant bacterial strains used or created in this study are listed at https://doi.org/10.6084/m9.figshare.7040249 (Table S1). Archived stocks of bacteria were maintained in 25% glycerol at −80°C. Prior to manipulations or experiments, bacteria were directly inoculated into 5 ml lysogeny broth (LB) media (10 g/liter NaCl, 5 g/liter yeast extract, 12 g/liter tryptone, 1 g/liter glucose) and grown for ∼16 h (overnight) with shaking at 30°C, except for E. coli HS, which was grown at 37°C. For growth on solid media, tryptic soy agar (TSA) was used. Gentamicin (10 μg/ml) was used to select recombinant strains tagged with the Tn*7* transposon, which was modified to carry a gentamicin resistance gene. When selecting merodiploid intermediates made using pAX1 or pAX2, which carry resistance to both gentamicin and chloramphenicol, either 10 μg/ml gentamicin or 5 μg/ml chloramphenicol was used. Selection of rifampin-domesticated variants was done using 100 μg/ml rifampin.

### Molecular techniques and reagents.

E. coli strains used for molecular cloning and conjugation and plasmids used or created during this work are listed at https://doi.org/10.6084/m9.figshare.7040315 (File S1). E. coli bacteria were typically grown in 5 ml LB liquid media at 30°C or 37°C with shaking in the presence of appropriate antibiotic selection to maintain plasmids. For propagation on solid media, LB agar was used. The antibiotic concentrations used were as follows: 100 μg/ml ampicillin, 20 μg/ml chloramphenicol, 10 μg/ml gentamicin, and 10 μg/ml tetracycline. A list of all DNA primers used for PCR, which are organized based on their “Wiles Primer” (WP) numbers, is provided at https://doi.org/10.6084/m9.figshare.7040315 (File S1). Unless specifically noted, standard molecular techniques were applied, and reagents were used according to the instructions of the manufacturers. Restriction enzymes and other molecular biology reagents for PCR and nucleic acid modifications were obtained from New England BioLabs (Ipswich, MA). Various kits for plasmid and PCR amplicon purification were obtained from Zymo Research (Irvine, CA). A Wizard genomic DNA purification kit (Promega, Madison, WI) was used for isolating bacterial genomic DNA. DNA oligonucleotides for PCR were synthesized by Integrated DNA Technologies (Coralville, IA). Sanger sequencing was done by Sequetech (Mountain View, CA). Custom gene synthesis was done by GenScript (Piscataway, NJ). A Leica MZ10 F fluorescence stereomicroscope with 1.0×, 1.6×, and 2.0× objectives and a Leica DFC365 FX camera were used for screening and imaging fluorescent bacterial colonies (Leica, Wetzlar, Germany). Images were captured and processed using standard Leica Application Suite software and ImageJ (71). Nucleotide sequences for putative *attTn7* sites are provided at https://doi.org/10.6084/m9.figshare.7040261 [File S2]) and were identified within *glmS* homologs that were manually retrieved from the Integrated Microbial Genomes & Microbiome Samples (IMG/M) website (https://img.jgi.doe.gov/m/) (72). Alignment of *attTn7* sequences was done using Clustal Omega (73) and visualized using the MView applet. Nucleotide sequences of 16S rRNA genes used for phylogenetic analysis are provided at https://doi.org/10.6084/m9.figshare.7040261 [File S2]) and were obtained via IMG/M or the RNAmmer Web tool (74). For *Variovorax* ZNC0006, the 16S rRNA gene was amplified and sequenced with primers 8F (AGAGTTTGATCCTGGCTCAG) and 1492R (GGTTACCTTGTTACGACTT) (75). 16S rRNA sequences were aligned using Clustal Omega, and the phylogenetic tree shown in Fig. 7 was generated using Dendroscope (76).

### Plasmid construction.

The plasmid-based tools developed in this work, along with their sequences, have been deposited at Addgene (Cambridge, MA) (https://www.addgene.org/). https://doi.org/10.6084/m9.figshare.7040315 (File S1) contains annotated nucleotide sequences of select genetic parts that were used to build plasmids and gene expression scaffolds. Specific details on how the plasmids were constructed are provided at https://doi.org/10.6084/m9.figshare.7040315 (File S1).

### Domestication-free Tn*7* tagging using pTn7xTS and pTn7xKS.

A detailed Tn*7* tagging protocol based on the use of pTn7xTS and pTn7xKS—including optimization and troubleshooting steps and notes on strain-specific procedures—is provided at https://doi.org/10.6084/m9.figshare.7040258 (Protocol S1). Generally, and as outlined in Fig. 1, triparental conjugation was performed between a single target bacterial strain, an E. coli SM10 donor strain carrying the transposase-containing pTNS2 helper plasmid, and an E. coli SM10 donor strain carrying either a pTn7xTS or pTn7xKS domestication-free tagging vector. Prior to mating, bacteria were prepared by subculturing them to an approximate optical density of 0.4 to 0.6 at 600 nm in LB media with the required antibiotics and at the appropriate growth temperature. Cells were then combined 1:1:1 (500 μl each), washed once by centrifugation and aspiration in 1 ml LB media or 0.7% NaCl, and suspended in a final 25-μl volume of the same medium as was used for washing. Next, the concentrated mating mixture was transferred to a 25-mm-wide 0.45-μm-pore-size filter disc (EMD Millipore, Billerica MA; product no. HAWP02500) that had been placed on top of a TSA plate. Once the mating mixture had dried, the plate was incubated at 30°C for 3 to 5 h. After incubation, the filter disc was placed in 1 ml 0.7% NaCl within a 50-ml conical tube and bacteria were dislodged by vortex mixing and pipetting. In cases where a pTn7xTS-based vector was used, 100 μl of the bacterial suspension was spread onto a TSA plate containing gentamicin and incubated overnight at 37°C to select for recombinants. To ensure the recovery of low-frequency recombinants, the remaining 900 μl of the suspension was pelleted by centrifugation, suspended in 100 μl 0.7% NaCl, and plated in the same way. In cases where a pTn7xKS-based vector was used, 100 μl of the bacterial suspension was spread onto a TSA plate containing gentamicin and 1 mM isopropyl-β-d-thiogalactopyranoside (IPTG) and incubated overnight at 30°C. The remaining 900 μl was prepared as described above, plated on TSA with gentamicin and IPTG, and incubated at 30°C.

The following day, putative recombinant target bacteria were subjected to colony purification by streaking on TSA without antibiotic selection at 30°C. Of note, when recombinant bacteria are tagged with a gene encoding a fluorescent protein, performing colony purification in the absence of antibiotic selection followed by visual screening of fluorescence is a convenient way to verify that the Tn*7* transposon has chromosomally integrated and the tagging vector has been lost. Purified clones were picked, cultured in LB media containing gentamicin, and genotyped by PCR to verify correct insertion of the Tn*7* transposon at the *attTn7* site. The universal primer WP11, which anneals within the Tn*7*R region of the Tn*7* transposon, was used with a species-specific primer that anneals to an adjacent chromosomal sequence within the 3′ end of the *glmS* gene to generate a small (∼250-bp) amplicon when the transposon is present. Species-specific primers used were as follows: *Vibrio* ZOR0018, WP50; *Vibrio* ZOR0035, WP51; *Vibrio* ZOR0036, WP12; *Vibrio* ZWU0020, WP12; *Aeromonas* ZOR0001, WP49; *Aeromonas* ZOR0002, WP52; *Pseudomonas* ZWU0006, WP256; *Acinetobacter* ZOR0008, WP259; *Enterobacter* ZOR0014, WP257; *Plesiomonas* ZOR0011, WP260; *Variovorax* ZNC0006, WP297; E. coli HS, WP150; *Aeromonas* HM21, WP49; *Shewanella* MR-1, WP48.

### Generation of markerless deletions via allelic exchange using pAX1 and pAX2.

A detailed protocol for carrying out allelic exchange using pAX1 and pAX2—which includes optimization and troubleshooting steps and notes on strain-specific procedures—is provided at https://doi.org/10.6084/m9.figshare.7040264 [Protocol S2]). Briefly, and as summarized in Fig. 3, allelic exchange cassettes for mediating markerless deletion of target genetic loci (i.e., the *pomAB* loci of *Vibrio* ZWU0020 and *Aeromonas* ZOR0001) were generated through splicing by overlap extension and inserted into a pAX-based vector. Next, diparental conjugation was performed between a single target bacterial strain (i.e., *Vibrio* ZWU0020 or *Aeromonas* ZOR0001) and an E. coli SM10 donor strain carrying the assembled allelic exchange vector. Prior to mating, bacteria were prepared by subculturing them to an approximate optical density of 0.4 to 0.6 at 600 nm in LB media with required antibiotics and at the appropriate growth temperature. Cells were then combined 1:1 (750 μl each), washed once by centrifugation and aspiration in 1 ml LB media or 0.7% NaCl, and suspended in a final 25-μl volume of the same medium as was used for washing. Next, the concentrated mating mixture was transferred to a 25-mm-wide 0.45 μm-pore-size filter disc that had been placed on top of a TSA plate. Once the mating mixture had dried, the plate was incubated at 30°C for 3 to 5 h. After incubation, the filter disc was placed in 1 ml 0.7% NaCl within a 50-ml conical tube and bacteria were dislodged by vortex mixing and pipetting. For the generation of *Vibrio* ZWU0020 Δ*pomAB*, which employed a pAX1-related vector, 100 μl of the bacterial suspension was spread onto a TSA plate containing gentamicin and incubated overnight at 37°C to select for merodiploids. The remaining 900 μl of the suspension was pelleted by centrifugation, suspended in 100 μl 0.7% NaCl, and plated in the same way to ensure recovery of rare recombinants. For the generation of *Aeromonas* ZOR0001 μ*pomAB*, which employed a pAX2-based vector, 100 μl of the bacterial suspension was spread onto a TSA plate containing gentamicin and 10 ng/ml anhydrotetracycline (aTc) and was incubated overnight at 30°C. The remaining 900 μl was prepared as described above, plated on TSA with gentamicin and aTc, and incubated at 30°C.

The following day, colonies of putative merodiploid target bacteria that were expressing the GFP tracker were purified by streaking on TSA without antibiotic selection at 30°C. This purification step also served to verify that the allelic exchange vector had integrated into the chromosome. Purified clones were picked, cultured in LB media containing gentamicin to maintain their merodiploid state, and archived as a frozen stock. To screen for second recombination events, merodiploids were cultured overnight in LB media without antibiotic selection and spread onto several TSA plates, again without antibiotic selection, at a density that allowed ∼100 to 200 discrete colonies to form. Colonies exhibiting partial or complete loss of GFP expression were purified by streaking on TSA at 30°C. Putative mutants were screened and genotyped by PCR using primers that flanked the modified locus, which produced two differently sized amplicons that represented the wild-type and mutant alleles. Primers WP71 and CheA2.ZW20.KOconfirm.REV were used to genotype *Vibrio* ZOR0035 Δ*cheA* mutants, primers WP163 and WP164 were used to genotype *Vibrio* ZWU0020 Δ*pomAB* mutants, and primers WP192 and WP195 were used to genotype *Aeromonas* ZOR0001 Δ*pomAB* mutants.

### In vitro growth measurements.

*In vitro* growth of bacterial strains was assessed using a FLUOstar Omega microplate reader (BMG Labtech, Offenburg, Germany). Prior to growth measurements, bacteria were grown overnight in 5 ml LB media at 30°C with shaking. Cultures were diluted 1:100 into fresh LB media and dispensed in quadruplicate (i.e., four technical replicates) (200 μl/well) into a sterile 96-well clear flat-bottom tissue culture-treated microplate (Corning, Corning, NY; product no. 3585). Absorbance measurements at 600 nm were then recorded every 30 min for 16 h (or until the stationary phase was reached) at 30°C with shaking. Growth measurements were repeated at least two independent times for each strain (i.e., two biological replicates) with consistent results. Data were exported and graphed using GraphPad Prism 6 software.

### Swim motility assays.

Prior to the assessment of swimming motility, bacteria were grown overnight in 5 ml LB media at 30°C with shaking. One milliliter of bacterial culture was then washed by centrifugation of cells at 7,000 × *g* for 2 min, aspiration of media, and suspension in 1 ml 0.7% NaCl. This centrifugation/aspiration wash step was repeated once more, and bacteria were suspended in a final volume of 1 ml 0.7% NaCl. One microliter of washed bacterial culture was then inoculated into a TSA plate containing 0.2% agar (30 g/liter tryptic soy broth and 2 g/liter Bacto agar). Swim plates were incubated at 30°C for 5 to 7 h and imaged on a Gel Doc XR+ imaging system (Bio-Rad, Hercules, CA). Motility assays were repeated at least two independent times (i.e., two biological replicates) with consistent results.

### Spot tests.

E. coli SM10 donor cells carrying vectors that contained temperature- and/or kill switch-based postconjugation counterselection systems were grown overnight in LB media with required antibiotics and at the appropriate growth temperatures. For assessing temperature-based counterselection, 10-fold serial dilutions were made on TSA plates containing gentamicin and incubated overnight at 30°C or 37°C. For assessing kill switch-based counterselection, 10-fold serial dilutions were made on TSA plates containing gentamicin with or without 1 mM IPTG (in the case of pTn7xKS) or 10 ng/ml aTc (in the case of pAX2) and incubated overnight at 30°C. Plates were imaged on a Bio-Rad Gel Doc XR+ imaging system. All spot tests were performed at least two independent times (i.e., two biological replicates), each including at least two technical replicates, with consistent results.

### Live imaging.

Live larval zebrafish were imaged using a home-built light sheet fluorescence microscope described in detail elsewhere (58, 77). In brief, a thin sheet of laser light is obtained by rapidly scanning the excitation beam with a galvanometer mirror. Fluorescence emission is captured by an objective lens mounted perpendicularly to the sheet. Three-dimensional images are obtained by translating the sample along the detection axis. The entire volume of the intestine (approximately 1,200 by 300 by 150 microns) is imaged in four subregions that are computationally registered after acquisition. The total time of acquisition of a single intestine is less than 1 min with 1-micron steps between planes. For all images, the exposure time was 30 ms and the excitation laser power was 5 mW prior to entry into the imaging chamber. Bacterial abundances were estimated using the analysis pipeline described in reference 58. Briefly, planktonic cells and multicellular aggregates were identified separately, and the number of cells per aggregate was estimated by dividing the total fluorescence intensity of the aggregate by the average intensity of planktonic cells for each strain.
